# Combined nitrogen and drought stress leads to overlapping and unique proteomic responses in potato

**DOI:** 10.1007/s00425-023-04085-4

**Published:** 2023-02-16

**Authors:** Katharina Wellpott, Anna M. Jozefowicz, Philipp Meise, Annegret Schum, Sylvia Seddig, Hans-Peter Mock, Traud Winkelmann, Christin Bündig

**Affiliations:** 1 Department of Woody Plant and Propagation Physiology, Institute of Horticultural Production Systems, Leibniz University Hannover, Herrenhäuser Straße 2, 30419 Hannover, Germany; 2 Applied Biochemistry, Department of Physiology and Cell Biology, Leibniz Institute of Plant Genetics and Crop Plant Research (IPK), OT Gatersleben, Corrensstr. 3, 06466 Seeland, Germany; 3 Institute for Resistance Research and Stress Tolerance, Julius-Kühn-Institute (JKI), Bundesforschungsinstitut Für Kulturpflanzen, Rudolf-Schick-Platz 3a, 18190 Sanitz, Germany; 4 Present Address: Universidad de Costa Rica, CIGRAS, 11501-2060 San Pedro, Costa Rica

**Keywords:** Abiotic stress, Combined stress, Label-free quantification, LC–MS, Protease, *Solanum tuberosum*, Stress response

## Abstract

**Main conclusion:**

Nitrogen deficient and drought-tolerant or sensitive potatoes differ in proteomic responses under combined (NWD) and individual stresses. The sensitive genotype ‘Kiebitz’ exhibits a higher abundance of proteases under NWD.

**Abstract:**

Abiotic stresses such as N deficiency and drought affect the yield of *Solanum tuberosum* L. tremendously. Therefore, it is of importance to improve potato genotypes in terms of stress tolerance. In this study, we identified differentially abundant proteins (DAPs) in four starch potato genotypes under N deficiency (ND), drought stress (WD), or combined stress (NWD) in two rain-out shelter experiments. The gel-free LC–MS analysis generated a set of 1177 identified and quantified proteins. The incidence of common DAPs in tolerant and sensitive genotypes under NWD indicates general responses to this stress combination. Most of these proteins were part of the amino acid metabolism (13.9%). Three isoforms of S-adenosyl methionine synthase (SAMS) were found to be lower abundant in all genotypes. As SAMS were found upon application of single stresses as well, these proteins appear to be part of the general stress response in potato. Interestingly, the sensitive genotype ‘Kiebitz’ showed a higher abundance of three proteases (subtilase, carboxypeptidase, subtilase family protein) and a lower abundance of a protease inhibitor (stigma expressed protein) under NWD stress compared to control plants. The comparably tolerant genotype ‘Tomba’, however, displayed lower abundances of proteases. This indicates a better coping strategy for the tolerant genotype and a quicker reaction to WD when previously stressed with ND.

**Supplementary Information:**

The online version contains supplementary material available at 10.1007/s00425-023-04085-4.

## Introduction

Potato (*Solanum tuberosum* L.) is one of the most important crops worldwide with a production of 359 million tons in 2020 (FAO 2020). In addition to table potato as a food source, starch potatoes are grown for industrial purposes such as paper, adhesives, or bioplastics due to their high starch content (Röper 2002).

With the growing world population and an increase in extreme weather conditions due to climate change, there is an urgent need to improve potato genotypes to ensure stable yields. Abiotic stresses, such as drought, are climate change-related problems in agriculture. In potato, such stresses can result in reduced plant growth and poor tuber yield and quality (Aliche et al. 2018; Hill et al. 2021). Due to their shallow root system, potato plants are more susceptible to drought stress than other crops. Therefore, irrigation is mostly essential for optimal yield (Zarzyńska et al. 2017). Furthermore, potato yield depends highly on sufficient N in the soil. N fertilization is unavoidable during periods of high vegetative growth in spring and early summer (Bélanger et al. 2000). Especially on sandy soils, where potatoes are mainly cultivated, the risk of N loss is high. Since the irrigation and fertilization phases fall into the same period and potato plants only take up 30–60% of the fertilized N from the soil, a high risk arises that N in form of nitrate (NO_3_^−^) leaches into the groundwater (Zerbarth and Rosen 2007). Therefore, N-efficient and drought-tolerant potato genotypes could mitigate these ecological problems and would be highly desired by farmers and breeders.

In the past, many transcriptomic studies have been performed to display the plant response to high and low levels of N as well as to drought stress. They demonstrated that numerous biological processes, such as amino and nucleic acid synthesis, protein folding, RNA processing, secondary metabolism and hormone biosynthesis are rapidly affected when nitrate is depleted or resupplied (Wang et al. 2003; Scheible et al. 2004; Gutiérrez et al. 2007). Carbohydrate metabolism, lipid metabolism, heat shock proteins and secondary metabolism are affected under drought stress (Evers et al. 2010; Aliche et al. 2022). In proteomic and transcriptomic studies on individual abiotic stressors such as salt (Legay et al. 2009), heat (Hancock et al. 2014), drought (Vasquez-Robinet et al. 2008; Boguszewska-Mankowska et al. 2020), or N deficiency (Jozefowicz et al. 2017; Meise et al. 2017; Tiwari et al. 2020a, 2020b) proteins and genes involved in the stress response were identified. Boguszewska-Mankowska et al. (2020) detected proteins that could be assigned to carbohydrate or amino acid metabolism to appear in higher abundance under drought stress conditions in a proteomic approach. Moreover, Vasquez-Robinet et al. (2008) found chaperones in a higher abundance under drought stress. Under N deficiency, Tiwari et al. (2020b) presented genes of protease inhibitors upregulated in a N-efficient potato cultivar. When abiotic stressors like drought and heat were applied in combination, evidence for divergently affected metabolic pathways and proteins was reported (Mittler 2006; Pandey et al. 2015; Demirel et al. 2020). However, knowledge about metabolic pathways and specific proteins for the combined stress of water deficit and N deficiency is absent for potato.

This study aimed to identify differentially abundant proteins (DAPs) in control and stress treatments to highlight general biochemical responses of potato to combined stress (NWD) as well as specific responses of genotypes with differing tolerance level to the provided stresses. This intended to get a deeper insight into the processes of abiotic stress tolerance and lead to identification of marker proteins. We chose a comprehensive proteomic approach to decipher the final metabolic adjustments rather than initial cellular responses. To pursue this aim, we selected two varieties, ‘Tomba’ and ‘Kiebitz’, among others, showing specific and contrasting reaction to either single or combined stress. With particular consideration of NWD, we showed both, general proteomic responses observed in both analyzed genotypes and divergent genotype dependent reactions to NWD. Differentially affected metabolic pathways were identified and related to the level of genotypes’ stress tolerance. Moreover, we emphasized differences in the responses to NWD as compared to the reactions to N deficiency (ND) and drought stress (WD).

## Materials and methods

### Plant materials

**Fig. 1:**
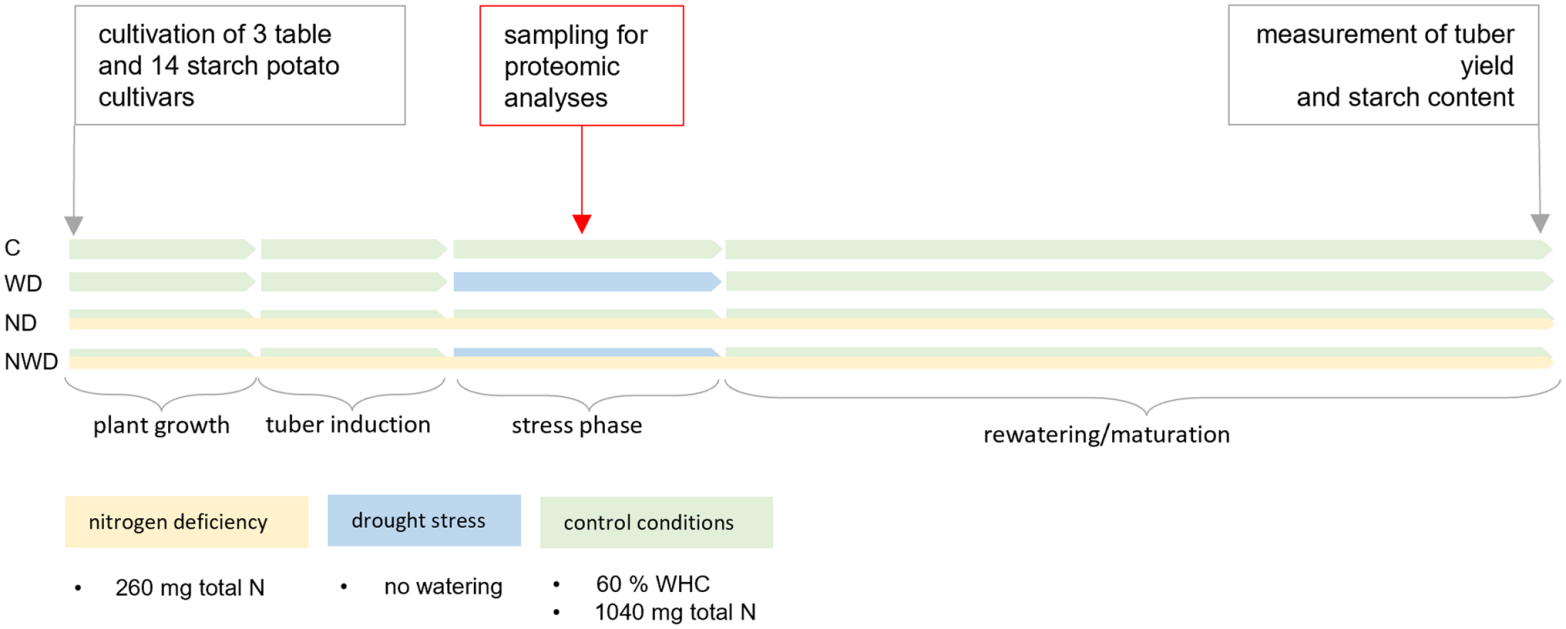
Timeline of pot trials with drought stress and N deficiency. Three table and 14 starch potato genotypes were cultivated until tuber formation (ND + NWD: 260 mg total N; C (control) and WD: 1040 mg total N). ND and C treatment received water up to 60% WHC, WD and NWD were not watered. At the first sign of wilting (5 d after stress onset), samples for proteomic analyses were taken. After the stress period, all plants were rewatered until maturity (60% WHC)

Plant material used for this study was sampled from two experiments in a rain-out shelter which took place in the Federal Research Centre for Cultivated Plants, Institute for Resistance Research and Stress Tolerance, Julius Kühn-Institute (JKI), Sanitz, Germany, in 2013 and 2015. Among 14 starch potato cultivars and 3 table potato cultivars tested in these experiments, the most divergently responding cultivars (hereafter: genotypes) ‘Eurostarch’, ‘Kiebitz’, ‘Kolibri’, and ‘Tomba’ were selected for this study based upon tuber and starch yield (Meise et al. 2019). Plants were grown under N deficiency (ND, supplied with a total of 260 mg N) and control conditions (C, cultivated at a continuous 60% water capacity, supplied with a total of 1040 mg N). Drought stress was applied at the beginning of tuber initiation. For this purpose, plants were kept 12–13 days without watering (WD), while the control treatment received water to maintain 60% water capacity during the whole experiment. Combined stress included both, drought and N deficiency (NWD). Details of the experimental setup are described in Meise et al. (2018, 2019) and an outline is given in Fig. 1. Samples were taken 5 days after drought stress initiation. The fourth and fifth leaflets of the youngest fully developed pinnate leaf were sampled and immediately frozen in liquid nitrogen (LN). Samples were stored at − 80 °C until analysis. Based on the calculation of the SSI (stress susceptibility index after Fischer and Maurer 1978) of the tuber and starch yield at harvest, the genotype ‘Tomba’ was found to be more tolerant to both, drought stress and N deficiency, as well as the stress combination in comparison to all other genotypes of the test set. The genotype ‘Kiebitz’ was found to be more sensitive under both stress situations compared to the other genotypes of the test set. Therefore, we will refer to the genotype ‘Tomba’ as ‘tolerant’ and to the genotype ‘Kiebitz’ as ‘sensitive’ hereafter. The genotypes ‘Eurostarch’ and ‘Kolibri’ showed contrasting responses depending on the stress type: ‘Eurostarch’ was assigned to the more tolerant genotypes, according to the SSI based on tuber yield under ND, whereas ‘Kolibri’ belonged to the more sensitive genotypes under NWD (Meise et al. 2019).

### Protein extraction and digestion

The protein processing and measurement were performed separately for single stress treatments ND and WD and the combination of stresses NWD. Control condition samples were measured in both analyses.

Frozen plant material was ground to a fine powder under LN using a mixer mill (MM400, Retsch, Haan, Germany; steel beads Ø 3 mm). A maximum of 100 mg of ground material was used for protein extraction. Leaf proteins were extracted using a trichloroacetic acid/acetone protocol (Tsugita and Kamo 1999) with some modifications. TCA (trichloroacetic acid) solutions A and B contained 20 mM DTT (dithiothreitol) instead of 0.07% 2-mercaptoethanol. The resulting dried pellets (25–35 mg) were resuspended in 100 µl lysis buffer (7 M urea, 2 M thiourea, 2% (w/v) CHAPS (3-((3-cholamidopropyl) dimethylammonio)-1-propanesulfonate), 5 mM DTT; pH 8.0), incubated for 1 h at 37 °C and centrifuged for 15 min at 17.000 g.

The concentration of protein in the solution was estimated using a 2-D Quant Kit (GE Healthcare, Munich, Germany) as previously described by Jozefowicz et al. (2020). Aliquots containing 10 µg of proteins were subjected to filter-based digestion, following Jozefowicz et al. (2017), which consisted of overnight digestion at 37 °C in a 1:50 dilution of sequencing grade modified trypsin (Promega, Mannheim, Germany). Before LC–MS analysis, peptides were suspended in 50 µl of 2% acetonitrile (ACN) and 0.1% (v/v) formic acid (FA).

### Label-free quantification of proteins

Peptides were analyzed by LC–MS, using Dionex UltiMate™ 3000 RSLCnano System (Thermo Fisher Scientific, Dreieich, Germany) coupled with an Impact II (Bruker Daltonics, Bremen, Germany). Digested protein samples were separated using a nano trap column (Acclaim PepMap100 C18, 5 μm, 100 Å) and an analytical column (Acclaim PepMap RSLC C18, Thermo Fisher Scientific, 50 cm × 75 µm).

600 µg of peptides were separated through a 2–40% acetonitrile gradient (buffer A: 0.1% FA in LC–MS grade water; buffer B: 0.1% FA in LC–MS grade ACN) over 120 min applying a flow rate of 300 nl/min. Due to loading and washing steps, the total time for an LC–MS/MS run was prolonged to 160 min.

The CaptiveSpray ion source with a nanoBooster device was used to connect the LC system to the MS instrument. The source was operated in positive ion mode at 150 °C dry temperature, 1300 V capillary voltage, 0.2 bar nanoBooster, and a dry gas flow of 0.3 l/min. For the MS and MS/MS acquisition, the predefined ‘Instant Expertise’ method was used (Compass 1.9, Bruker). Briefly, the *m/z* data were acquired in the range of 150 to 2200 and the fixed total cycle time was set to 3.0 s. The instrument settings were as follows: hexapole radio frequency (RF) voltage of 350 V peak-to-peak (Vpp), a funnel 1 RF of 400 Vpp, a funnel 2 RF of 600 Vpp, a pre-pulse storage time of 10 μs, a transfer time of 90 μs and a collision cell RF of 2000 Vpp. For the MS spectra, the acquisition speed was 2 Hz with a collision energy of 7 eV. For the MS/MS, the acquisition speed was dependent on the precursor signal intensities and was set to 4 Hz for lower (2500 cts) and 16 Hz for higher (25,000 cts) intensities with linear adjustment for the precursors between low and high. The collision energy was adjusted between 23 and 65 eV as a function of the *m*/*z* value. The instrument was calibrated using 10 mM sodium formate.

### Data analysis

The acquired spectra were processed for label-free quantifications using Progenesis QI software for proteomics (Version 3.0, Nonlinear Dynamics, Newcastle upon Tyne, UK) as recommended by the manufacturer, thereby enabling mass correction, alignment, normalization, peak picking, quantification, and statistics. MS/MS spectra were exported from the Progenesis QI software as Mascot generic files and used for peptide identification with Mascot v2.5.1. The potato database based on the sequences from *Solanum tuberosum* group Phureja DM1-3 (PGSC_DM_v3.4_pep_representative, 39,031 entries) (Xu et al. 2011) was annotated by matching against available NCBI entries with Blast2GO software (09.2014) (Conesa and Götz 2008) and merged with the sequences of human keratin and trypsin. The search parameters applied were as follows: 15 ppm peptide mass tolerance, 0.05 Da fragment mass tolerance, one missed cleavage allowed, carbamidomethylation as fixed modification, and methionine oxidation as variable modification. A Mascot integrated peptide decoy database search was performed and searches were processed with the Percolator machine-learning algorithm (Käll et al. 2008). The false discovery rate was < 1% and ion score cut-off 13. For subsequent analysis, the set of identified sequences was re-imported into Progenesis QI. Quantification was performed for proteins identified with at least two unique peptides. The results of protein quantification were exported and further analyzed in MS Excel.

### Statistics and selection of differentially abundant proteins (DAPs)

The protein data obtained for experiment 1 (2013) and experiment 2 (2015) for each genotype were analyzed separately, due to the different weather conditions in both years, particularly very high temperatures during the 5 days of water withdrawal in experiment 1 (mean temperature 2013: 22.03 °C; 2015: 19.03 °C) (Meise et al. 2018). Only proteins that were of significantly changed abundance in both experiments (student’s *T* test *P* < 0.05 and fold change stress/control < 0.66 or > 1.5) were considered DAPs. Proteins of differential abundance in single experiments only were considered as altered due to additional factors such as fluctuations in the weather conditions and were therefore withdrawn from further analysis. Venn diagrams were created using Venny 2.0 tool (Oliveros 2007).

Additional annotation for selected proteins was sought by referring to the UniProt server (www.uniprot.org). Proteins were functionally classified according to KEGG orthology using BlastKoala or manual classification in case functions could not be assigned automatically. Principal component analysis, Z-score normalization, and hierarchical clustering based on the Euclidean distance method were carried out using the Perseus Framework (Tyanova et al. 2016). A full listing of the differentially expressed proteins has been archived, together with all of the raw data, in the IPK Gatersleben system e!DAL (Arend et al. 2014), available at: https://doi.org/10.5447/IPK/2023/4.

## Results and discussion

### Genotype ‘Tomba’ was selected as more tolerant to ND, WD, and NWD

**Fig. 2:**
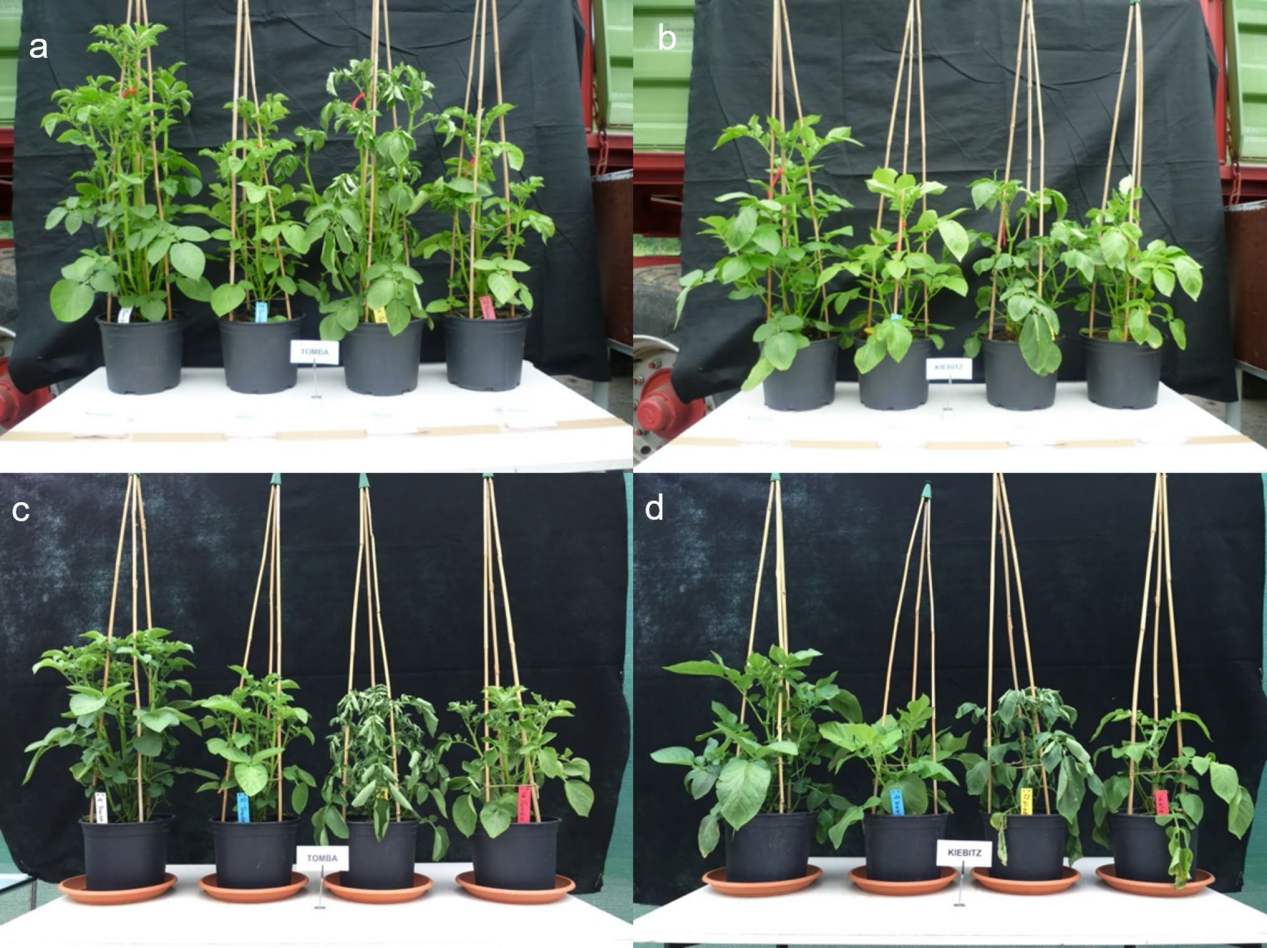
Condition of genotype ‘Tomba’ in control, ND, WD and NWD treatment (left to right) in experiment 1 (**a**) and experiment 2 (**c**), and of genotype ‘Kiebitz’ in experiment 1 (**b**) and 2 (**d**)

A previous study, in which the performance of 14 starch and 3 table potato genotypes was compared under N deficiency (ND), water deficiency (WD), and a combination of stresses (NWD) in two rain-out-shelter experiments (Meise et al. 2018), was the basis for the proteomic analysis in the present investigation. Out of the 14 starch genotypes, 4 genotypes with the most contrasting response to a combination of drought and N deficiency were selected. Genotype ‘Tomba’ exhibited the highest tuber yield under two of the three applied stress conditions (WD, NWD), whereas ‘Eurostarch’ had a slightly higher yield under N deficiency (ND). On the contrary, genotype ‘Kiebitz’ produced the lowest tuber biomass under control, N deficiency, and water deficiency conditions within the experiments. Genotype ‘Kolibri’ produced the lowest yield when combined N and water deficiency (NWD) was applied. When both stresses were combined, genotype ‘Kiebitz’ produced only 38%, whereas genotype ‘Tomba’ still produced 68% of the tuber fresh weight under control conditions (Meise et al. 2018). The changes in the growth (Fig. 2) and the nutritional status of the potato plants were displayed by measuring N_Kjeldahl_, total protein content, soluble sugars, and proline content upon stress application of the genotypes analyzed (Suppl. Table S1). ‘Tomba’ showed a higher N content with 30.8 ± 6.4 mg N/g DM than ‘Kiebitz’ with 19.5 ± 1.1 mg N/g DM after NWD. Pure protein content, as well as proline content, were also higher in ‘Tomba’ (26.7 ± 5.0 mg/g DM; 4.0 ± 3.2 µmol/g DM) than in ‘Kiebitz’ (18.9 ± 1.8 mg/g DM; 2.2 ± 0.7 µmol/g DM). Relative water content was 80.1 ± 3.8% for ‘Tomba’, while it dropped to 76.1 ± 3.9% for ‘Kiebitz’. Plant height also differed between the two genotypes. With 16.9 ± 1.8 cm, ‘Kiebitz’ was the shortest genotype of the tested genotypes after combined stress. ‘Tomba’ reached a plant height of 22.7 ± 2.2 cm.

### Different numbers of proteins are changed in potato genotypes after NWD treatment

**Fig. 3:**
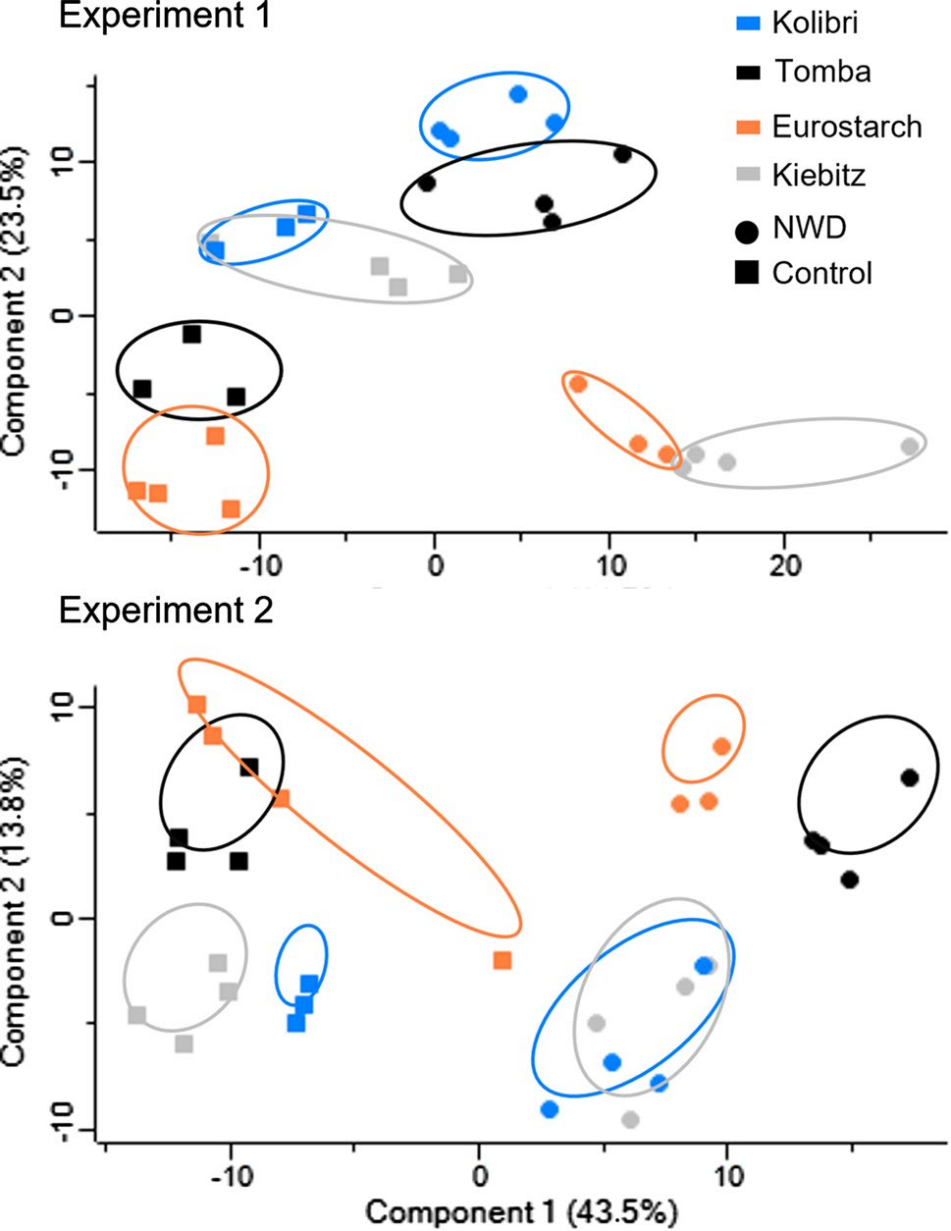
Principal component analysis showed a clear separation of the NWD treatment from controls for all four potato genotypes. The shape of data points indicates combined N and water deficiency (NWD, circle) or control conditions (square). The colors indicate the genotype: ‘Tomba’ (black), ‘Kiebitz’ (grey), ‘Eurostarch’ (orange), ‘Kolibri’ (blue)

The label-free LC-MS analysis generated a set of 1177 identified and quantified proteins, based on 6,060 non-conflicting peptides (the full list of identified proteins is stored together with raw data in the e!DAL system of the IPK-Gatersleben). The differences in the protein profiles were elucidated by a principal component analysis (PCA) for both experiments independently (Fig. 3). The four potato genotypes and the treatments were separated by PC1 and PC2, with PC1 accounting for 44.7% and 43.5% of the explained variance in experiments 1 and 2, respectively, and PC2 for 23.5% and 13.8%. The clustering of protein profiles showed differences in experiments 1 and 2. For instance, genotypes ‘Kiebitz’ and ‘Kolibri’ behaved similarly under control conditions in experiment 1 and under NWD treatment in experiment 2. Thus, additional factors (temperature, air humidity) might have influenced the protein profiles during both experiments (Georgii et al. 2017), and therefore, only proteins with significantly changed abundance in both years were considered differentially abundant proteins (DAPs). Applying this filter, for the NWD stress treatment 234, 199, 199, and 74 DAPs were identified in genotypes ‘Tomba’, ‘Eurostarch’, ‘Kiebitz’, and ‘Kolibri’, respectively. These will be discussed in detail in the following sections with emphasis on common (shared) general responses to NWD seen in all four genotypes, before the specific responses of the tolerant genotype ‘Tomba’ and the sensitive genotype ‘Kiebitz will be elaborated. Finally, the response to the combined stress will be compared to both single stresses, N deficiency and drought.

### General response of potato genotypes to NWD stress

**Fig. 4:**
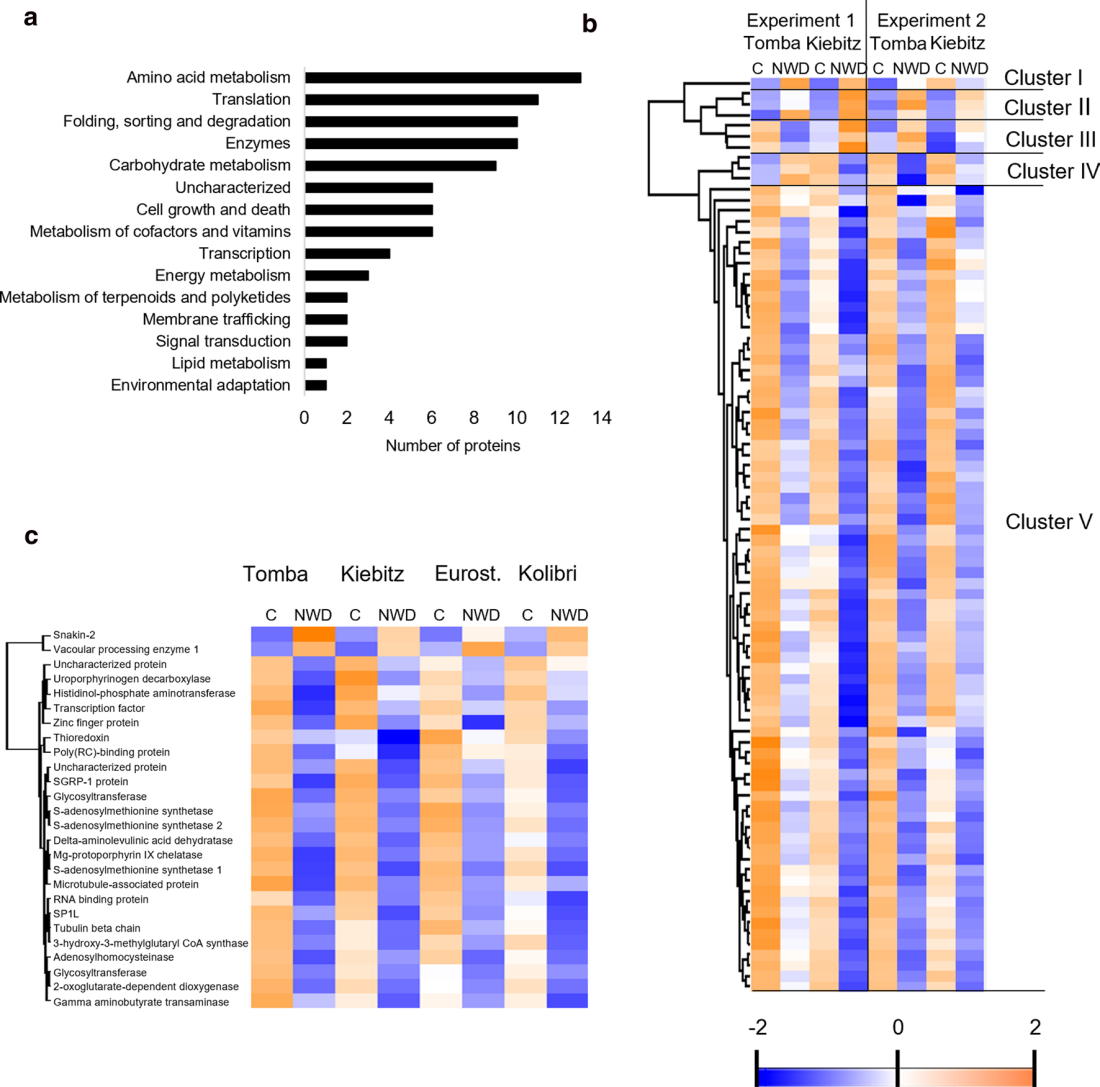
Eighty-six proteins showed a similar response to NWD stress in ‘Tomba’ and Kiebitz’.** a** Functional classification of proteins responding to NWD stress in both genotypes. The classification was performed according to the KEGG orthology. **b** Heat map representation of proteins with a similar response to NWD in ‘Tomba’ and ‘Kiebitz’ divided into five clusters with similar abundance profiles. Hierarchical clustering was carried out with k-means preprocessing and was based on average Euclidean distance linkage. Relative abundance in the heatmap has been color-coded following Z-score normalization. Each column represents one treatment. *NWD* N and water deficiency; *C* control conditions. **c** Heat map representation of proteins with a similar response to NWD in all four potato genotypes. Eurost, ‘Eurostarch’

The focus of this study was set on the comparison of DAPs in the genotypes ‘Tomba’ and ‘Kiebitz’, as these genotypes showed the most contrasting response to ND, WD, and NWD (Meise et al. 2019). Most of the proteins considered in the following paragraph showed a very similar trend in abundance alteration in the other two genotypes ‘Kolibri’ and ‘Eurostarch’ but did not fulfill the criteria of significance as stated in Material and methods (e.g. student’s T test *P* and FC stress/control, Suppl. Table S2). The comparison of DAPs in ‘Tomba’ and ‘Kiebitz’ revealed that 86 proteins were significantly changed in both genotypes, whereas 148 were specific to ‘Tomba’ and 113 to ‘Kiebitz’ (Suppl. Fig. S1). Functional assignment of the proteins according to KEGG orthology was performed to gain a first understanding of processes commonly regulated in response to NWD stress (Fig. 4a). The hierarchical clustering analysis revealed five clusters of DAPs with similar regulation in response to NWD stress (Fig. 4b, Suppl. Table S2). Only one DAP belonged to cluster I (ribosomal protein S10), which reacted with an increase in relative abundance to NWD in both genotypes in experiment 1 but decreased in ‘Kiebitz’ in experiment 2. Cluster II comprised three DAPs (vacuolar processing enzyme 1, snakin-2, and remorin) with an increase in abundance due to the NWD stress. Cluster III grouped three DAPs (cytochrome C oxidase polypeptide, cell wall invertase, and cysteine peptidase 3). Those proteins increased in the stress response, with the exception of ‘Tomba’ in experiment 1. Cluster IV captured three DAPs (ATP-dependent Clp protease, lysine- tRNA ligase, and ribulose bisphosphate carboxylase large chain), that decreased in the stress response, with exception of ‘Tomba’ in experiment 1. Finally, cluster V contained the majority of NWD-responding DAPs, which decreased in relative abundance in response to stress in both genotypes. The overrepresented processes and pathways in cluster V were: TCA cycle and glycolysis (fructose-bisphosphate aldolase, pyruvate dehydrogenase E1 and E2 component, diphosphate-dependent phosphofructokinase, dihydrolipoamide dehydrogenase, and pyruvate kinase), chlorophyll synthesis (Mg-protoporphyrin IX chelatase, delta-aminolevulinic acid dehydratase, uroporphyrinogen decarboxylase, protoporphyrinogen oxidase, glutamate-1-semialdehyde 2,1-aminomutase and glutaminase), ethylene biosynthesis (S-adenosylmethionine synthetase (SAMS)-3 isoforms, adenosylhomocysteinase, and aminocyclopropane carboxylate oxidase) and cytoskeleton proteins (Ase1/PRC1/MAP65 family protein, katanin p60 ATPase, tubulin alpha and beta). The 86 DAPs and their functional classification are accessible in detail in Suppl. Table S2, in the same order as presented in the heat map (Fig. 4b).

Amino acid metabolism was assigned the largest group of DAPs (12 DAPs; 13.9%), which differed significantly in abundance in both genotypes, especially cysteine and methionine metabolism. As many as three isoforms of SAMS were significantly less abundant under NWD in both genotypes and experimental years (Cluster V) (Suppl. Table S2). S-adenosyl-l-methionine (SAM) is a key metabolite for different processes such as polyamine biosynthesis as well as lignin biosynthesis and is catalyzed by SAMS from methionine and ATP. SAMS is also known to have a function in the response to environmental stressors (Heidari et al. 2020). Kim et al. (2015) were able to link SAMS in wild potato (*S.* *brevidens*) to the upregulation of ABA and ethylene metabolic pathway genes. They detected higher salt and drought stress tolerance when a full-length cDNA of SAMS from *S. brevidens* was overexpressed in *Arabidopsis thaliana*. Because SAM is the precursor for ethylene (Amir 2010), the expression of SAMS is important for ethylene biosynthesis and, therefore, for the regulation of growth and senescence (Khan et al. 2015). Zhang et al. (2020) reported overexpression of *SlSAMS1* to influence the reaction to drought, salt stress, low temperature, and ABA treatments in *Solanum lycopersicum*. They showed increased abiotic stress tolerance in *SlSAMS1-*overexpressing plants by improved water retention and photosynthesis capacity as well as higher levels of ROS-scavenging enzymes. In contrast, in our study, we found a lower abundance of SAMS in NWD. Furthermore, SAMS was also observed to be lower abundant in WD in all genotypes and ND in the more tolerant genotypes ‘Tomba’ and ‘Eurostarch’. ND was applied since the beginning of the experiment (5 weeks in total). Subsequently, 5 days before sampling of the leaves, water deficiency in the corresponding variants started. Due to the prolonged nitrogen deficiency, it could be assumed that components such as methionine were used up by the plants at the time of protein analysis. Methionine should be measured in a future trial to provide information on methionine content in potato leaves.

Ten DAPs out of the 86 DAPs under NWD were associated with folding, sorting, and degradation of proteins. Two proteins were found to be higher abundant in both genotypes after combined stress was applied (Cluster II): vacuolar processing enzyme (VPE) and cysteine proteinase 3-like. Both proteins are known to be key factors in programmed cell death and thus related to abiotic stress (Solomon et al. 1999; Teper-Bamnolker et al. 2021). While programmed cell death is a way for plants to selectively eliminate damaged cells and recycle nutrients (Wingler et al. 2004), a higher abundance of VPE and cysteine proteinase 3-like might be a strategy for the plant to cope with abiotic stresses. VPE is an enzyme that is stimulated by various stressors like heat, oxidative, and salt as well as biotic stressors. Besides being involved in PCD, vacuolar processing enzymes are also described to be responsible for processing protein precursors of chitinases and proteinase inhibitors to evoke their active forms (Yamada et al. 2020).

In general, abiotic stress reduces photosynthesis efficiency either directly due to decreased CO_2_ availability by stomatal closure or indirectly by oxidative stress (Chaves et al. 2009; Golldack et al. 2014). Therefore, plants are facing a reduced energy supply in form of C products during abiotic stress. Nine DAPs were identified, being associated with C metabolism. Especially proteins linked to glycolysis, the pentose phosphate pathway, and the TCA cycle were found to be less abundant under combined stress. Pyruvate dehydrogenase (PDH) is the first enzyme of the PDH complex, which enables the entry of C into the TCA cycle and thus energy production (Ohbayashi et al. 2019). With less PDH available, less carbon is fed into the TCA cycle, which is, therefore, unavailable for respiration. Thus, the higher abundance of cytochrome c oxidase subunit 6b, which was determined for ‘Kiebitz’ in both years and for ‘Tomba’ in 2015, might help to sustain respiration, as it is part of complex IV and a terminal electron acceptor of the mitochondrial respiratory chain (Chen et al. 2009). The increased respiration could be used to generate ATP for nutrient recycling and export during senescence and PCD (Mayta et al. 2019). Metabolic and functional studies would be needed in future studies to shed light on the amino acid and carbohydrate metabolism under combined stress.

Biosynthesis of special cofactors e.g. ascorbate (ABA biosynthesis) can be linked to abiotic stressors such as light and drought (Smith et al. 2007) as well as to drought stress tolerance in maize and soybean (Krannich et al. 2015). In our study, six DAPs were lower abundant in NWD and identified to be related to the metabolic pathways of cofactors and vitamins. Most of them were found to be associated with chlorophyll biosynthesis, which is essential for functional photosynthesis. This process was likely reduced under NWD stress as indicated by the lower abundance of porphobilinogen synthase (also 5-aminolevulinate dehydrogenase or delta-aminolevulinic acid dehydratase), which combines two molecules of 5-aminolevulinic acids to form porphobilinogen, and magnesium chelatase, which catalyzes the reaction of protoporphyrin IX to Mg-protoporphyrin IX in a later step (Ohmiya et al. 2014). This leads to less efficient photosynthesis, which has also been described in potato by Li et al. (2016) under NWD and by Aliche et al. (2018) under WD. Magnesium chelatase has also been linked to ABA-mediated signaling and ABA-induced stomatal closure. In *Arabidopsis thaliana* mutants, overexpressing the Mg-chelatase H subunit, a higher tolerance to drought stress was observed (Tsuzuki et al. 2013). In line with this observation, when Meise et al. (2017) applied ND in an in vitro test system, they found magnesium chelatase to be higher abundant after ND in a tolerant potato genotype. In the present study, however, magnesium chelatase was lower abundant in all genotypes under NWD.

### Proteins specific to the tolerant genotype ‘Tomba’

**Fig. 5:**
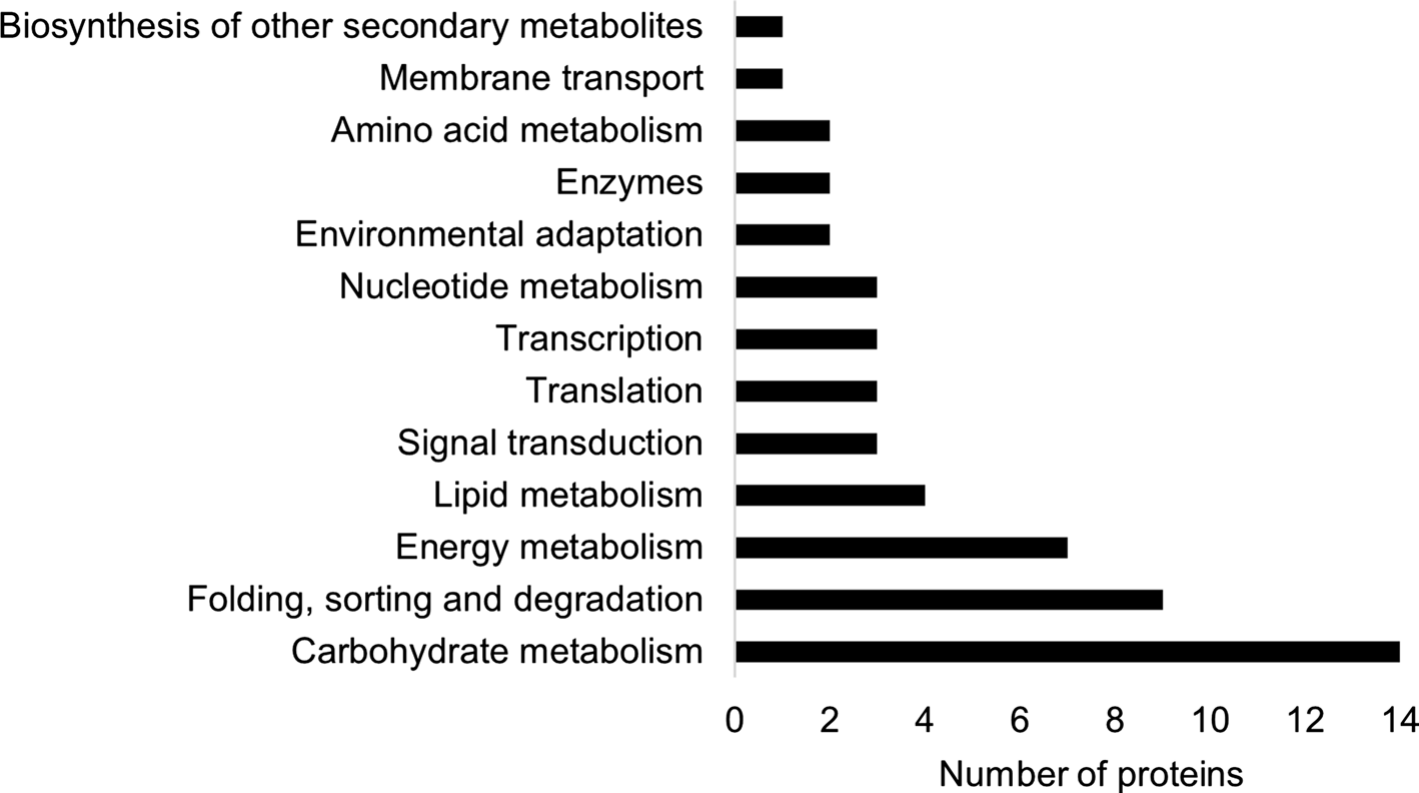
Functional categories of DAPs specific for the tolerant genotype ‘Tomba’. Most proteins responsive to NWD in the genotype ‘Tomba’ belonged to categories carbohydrate and energy metabolism. The classification was performed according to the KEGG orthology

148 proteins were differentially abundant only in the genotype ‘Tomba’ (Suppl. Table S3). The revision of the DAPs, however, showed that 93 of them displayed similar trends in the genotype ‘Kiebitz’ but did not meet the criteria of significance in one of the experiments (student’s T test *P* and FC stress/control). This is because drought stress started earlier in ‘Tomba’ than in ‘Kiebitz’ and there were high temperatures in experiment 1 when the plants were stressed (Meise et al. 2018). Consequently, the substrate dried faster in that experiment. Additionally, the substrate dried out more quickly in ‘Tomba’ pots due to their greater biomass. For that reason, those proteins should be rather considered a common response to NWD stress. We decided to exclude those proteins from further analysis, to focus on the DAPs differentially abundant only in the tolerant genotype. Among these remaining 55 DAPs specific to the genotype ‘Tomba’, 14 were assigned to the category carbohydrate metabolism (Fig. 5). Other overrepresented categories included protein folding, sorting and degradation (nine DAPs), energy metabolism (seven DAPs), and lipid metabolism (four DAPs).

**Fig. 6:**
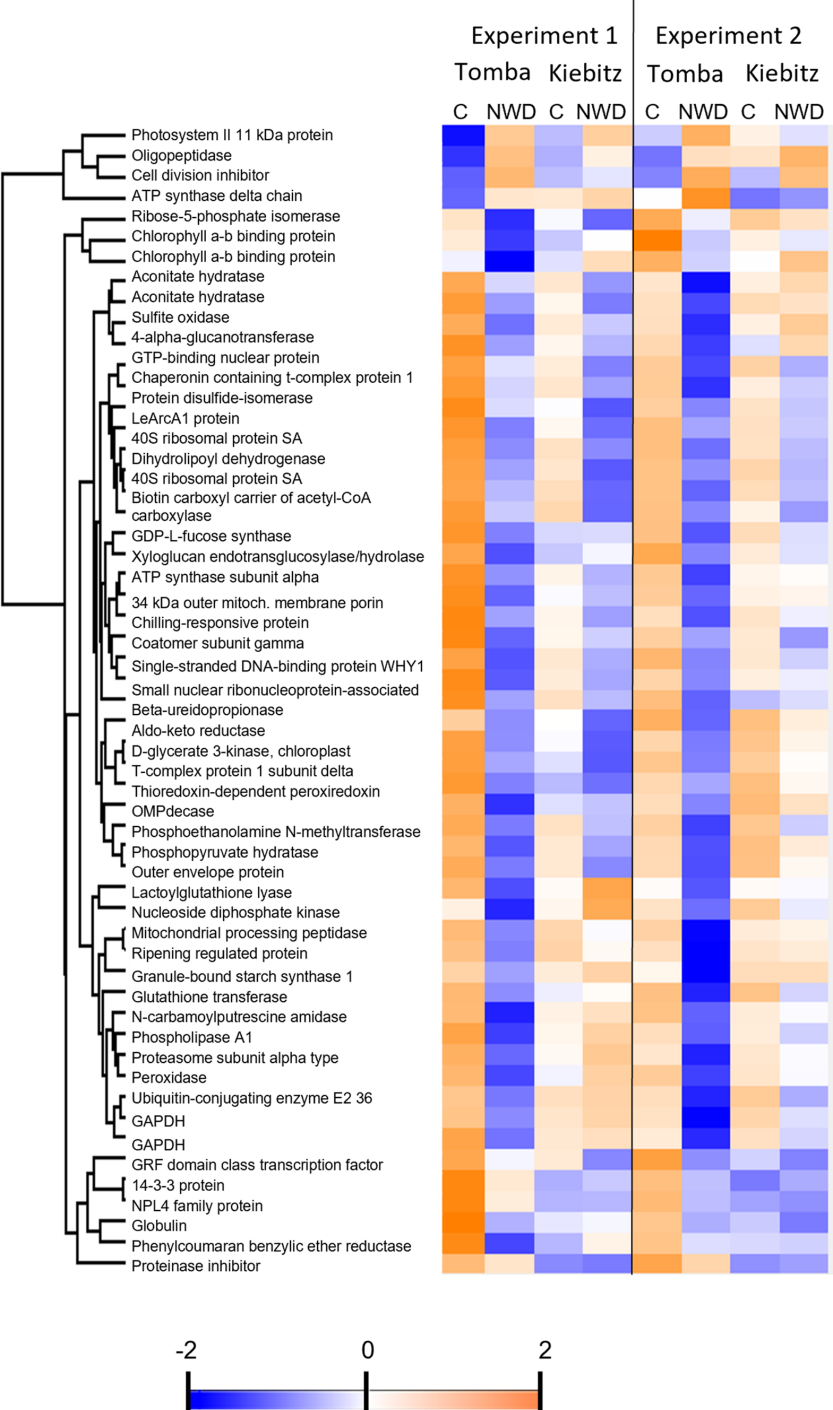
Fifty-five DAPs were specific to the tolerant genotype ‘Tomba’. Hierarchical clustering was carried out with k-means preprocessing and was based on average Euclidean distance linkage. Relative abundance in the heatmap has been color-coded following Z-score normalization. Each column represents one treatment. NWD, N and water deficiency

Only four of the 55 DAPs showed a higher abundance in the NWD treatment compared to the control (photosystem II 11 kDa protein, oligopeptidase, cell division inhibitor, and ATP synthase, Fig. 6). Three of them (except cell division inhibitor) were assigned to energy metabolism. One of the first responses to abiotic stress in plants is down-regulation of energy metabolism (Romero et al. 2017; Dahal et al. 2019). The fact that the tolerant genotype ‘Tomba’ contained proteins of energy metabolism in higher abundance may indicate that—after dealing with the stress—it was already able to upregulate its energy metabolism to return to a normal physiological state. However, this hypothesis cannot be verified with the current setup, as further earlier samples of N deficiency would have to be analyzed.

Interestingly, the lactoylglutathione lyase (synonyme: glyoxalase) was lower abundant under NWD stress in the genotype ‘Tomba’, but higher abundant in genotype ‘Kiebitz’ in experiment 1. Lactoylglutathione lyase regulates methylglyoxal, which is a cytotoxic compound inhibiting cell proliferation and leading to degradation of proteins, thus affecting the antioxidant defense system negatively (Upadhyaya et al. 2011). Because genotype ‘Tomba’ was categorized as tolerant to NWD based on the tuber yield and starch content, a lower abundance of lactoylglutathione lyase might help to maintain normal metabolism. Likewise, nucleoside diphosphate kinase was lower abundant under NWD in genotype ‘Tomba’, but higher abundant in genotype ‘Kiebitz’ in experiment 1. This protein is a housekeeping enzyme, which can be associated with ROS scavenging (Moon et al. 2003). Jozefowicz et al. (2017) presented an altered protein composition in potato roots under ND. Lactoylglutathione lyase was higher abundant in the tolerant genotype in the study of Jozefowicz et al. (2017), whereas in our study, the protein showed higher abundance in the sensitive genotype. These DAPs deserve further analysis involving earlier time points and gene expression analyses to determine their role in stress response.

Fourteen proteins (25.5%) were assigned to the functional group of carbohydrate metabolism. The metabolic pathways of the pentose phosphate pathway, glycolysis, and the TCA cycle were affected, but proteins of the starch metabolism were also less abundant after NWD. The aconitate hydratases from the TCA cycle and the glyoxylate cycle (Moeder et al. 2007) are cycle-maintaining proteins. The mitochondrial aconitate hydratase also provides 2-oxoglutarate for amino acid synthesis and ammonia assimilation (Araújo et al. 2014; Eprintsev et al. 2021). Due to the lower N availability in the NWD treatment, the lower abundance of this protein could indicate a stress response toward N deficiency. Also, in the tolerant genotypes ‘Eurostarch’ and ‘Tomba’, there was a lower abundance in both individually applied stresses.

Three proteins were assigned to nucleotide metabolism (nucleoside diphosphate kinase, OMPdecase, and beta-ureidopropionase). Proteins of the nucleotide metabolism are needed in several energetic reactions such as the TCA cycle (nucleoside diphosphate kinase), as well as in the de novo biosynthesis of pyrimidines (Witte and Herde 2020), which can be connected to the pentose phosphate pathway. Since pyrimidines also contain N, a lower abundance of related proteins could indicate this to be part of the N deficiency response.

Proteins and enzymes involved in proteolysis are responsible, amongst others, for the degradation of proteins (van Wijk 2015). Three proteins from the category folding, sorting, and degradation (ubiquitin-conjugating enzyme E2, proteasome subunit alpha, proteinase inhibitor) were identified to be less abundant under NWD stress compared to the control. This could indicate that ‘Tomba’, as a tolerant genotype, was able to adapt to N deficiency which had been applied since the beginning of the experiment. This genotype could better cope with the additional drought stress and thus protect its resources. Meise et al. (2019) showed similar levels of protein content under NWD stress and in the control treatment in genotype ‘Tomba’. Genotype ‘Kiebitz’ on the other hand showed lower protein contents in NWD than in the control (Meise et al. 2018). This might indicate, that genotype ‘Tomba’ decreased the proteolysis to maintain or return to a normal level of metabolism after the initial stress response.

### Proteins specific to sensitive genotype ‘Kiebitz’

In our proteomic analysis, 113 proteins significantly changed due to NWD stress in the sensitive genotype ‘Kiebitz’ (Suppl. Table S4). However, the number of DAPs decreased drastically when proteins, which showed significant differences in abundance in ‘Tomba’ in experiment 2 as well, were excluded. We could observe that the abundance of many proteins changed in the same way in ‘Kiebitz’ and ‘Tomba’ in experiment 2 but not in experiment 1. The differences were probably driven by additional high temperatures in the five-day drought stress treatment during experiment 1 as explained earlier (Meise et al. 2018). After the exclusion of those proteins, there were 19 DAPs highly specific to the more sensitive genotype (Table 1). Eleven of them increased in abundance in response to NWD stress, whereas eight showed a decrease.

**Table 1:** List of differentially abundant proteins in the sensitive potato genotype ‘Kiebitz’ induced by N deficiency combined with drought stress (NWD)

Accession	Protein description (according to Uniprot)	KEGG classification 2nd dimension	Fold change stress/control			
			Experiment 1		Experiment 2	
			Tomba	Kiebitz	Tomba	Kiebitz
400029393	Plasma membrane polypeptide	Signal transduction	1.33	1.54	1.44	1.74
400058896	Aldehyde dehydrogenase (NAD( +))	Carbohydrate metabolism	0.58	2.14	1.45	2.17
400078034	NAD(P)H dehydrogenase (quinone)	Metabolism of cofactors and vitamins	0.94	2.07	1.47	2.03
400065518	Peroxidase	Biosynthesis of other secondary metabolites	0.64	2.48	1.42	1.64
400046584	Aldose 1-epimerase	Carbohydrate metabolism	0.66	1.68	1.21	1.54
400088012	Subtilase family protein	Folding, sorting and degradation/signaling molecules and interaction	0.86	1.83	1.40	1.78
400031890	Purple acid phosphatase	Protein phosphatases and associated proteins	0.88	1.92	1.21	1.57
400009216	Alpha-mannosidase	Glycan biosynthesis and metabolism	0.73	1.99	0.91	1.70
400017451	Subtilase	Folding, sorting and degradation/signaling molecules and interaction	0.92	1.66	0.63	2.05
400066639	Carboxypeptidase	Peptidases and inhibitors	1.00	1.59	0.93	1.54
400081312	Pectinesterase	Carbohydrate metabolism	0.68	1.86	1.48	1.50
400039443	Plastid RNA-binding protein	Environmental adaptation	1.16	0.51	0.75	0.59
400016844	Pyruvate kinase	Carbohydrate metabolism	0.80	0.62	0.82	0.50
400026666	Assimilatory sulfite reductase	Energy metabolism	1.22	0.51	0.70	0.27
400051668	Poly(RC)-binding protein	Messenger RNA biogenesis	0.68	0.46	0.71	0.64
400057203	RNA Binding Protein 45	Transcription machinery/Messenger RNA biogenesis	0.79	0.47	0.69	0.62
400078506	Fruit protein PKIWI502	Signaling molecules and interaction	0.78	0.62	0.68	0.61
400026271	Stigma expressed protein	Peptidases and inhibitors	0.76	0.38	0.65	0.38
400055527	Single-stranded DNA binding protein	Ribosome biogenesis	0.68	0.63	0.68	0.54

The abundance is presented in the form of fold change. Accession numbers are given without the PGSC003DMT prefix. Full details of the protein identification are stored together with raw data

Among these DAPs, four proteins were assigned to the category of proteases/protease inhibitors. The three proteases (subtilase, carboxypeptidase, subtilase family protein) were higher abundant in the NWD treatment than in the control treatment. The protease inhibitor found in this study (stigma expressed protein) was less abundant in the NWD treatment. This protein showed a similar pattern of abundance in genotype ‘Kolibri’, which was also considered sensitive to NWD stress (Suppl. Table S4). Proteases are involved in diverse cellular processes such as photoinhibition in the chloroplast, defense mechanisms, PCD, and thus protein denaturation, which is triggered by different abiotic stresses, such as drought stress (Estelle 2001). Protease inhibitors can prevent the dismantling of proteins by proteases and their decreased abundance under abiotic stress can thus result in free N that can be used for recycling (Folgado et al. 2013). Thus, our results indicate that sensitive potato genotypes responded to NWD with increased protein degradation. During senescence and ND, proteases like subtilisin and the proteasome were reported to degrade soluble proteins and recycle RuBisCO in oilseed rape indicating a response specific to ND (Poret et al. 2019).

Four DAPs were assigned to the carbohydrate metabolism, of which three proteins [aldehyde dehydrogenase (NAD(+)), pectin esterase, aldose 1-epimerase] were higher abundant in the stress treatment. Aldehyde dehydrogenase (NAD(+)) is an initial stress response protein that occurs during water deficiency, N deficiency, and salt stress (Kirch et al. 2005; Meise et al. 2017) and supports the vegetative growth of the plants (Tola et al. 2020). It was also found in NWD in all other genotypes but only in 1 year (experiment 2, year 2015), which might suggest a common response mechanism to NWD stress among the genotypes. The peroxidase 3-like protein, which was higher abundant under NWD in this study, is also classified as a protein of the initial stress response. It is striking that this protein was altered only in the sensitive genotype. Pectin esterases are involved in cell wall formation, specifically in plasticity of the cell wall. A higher abundance of pectin esterase in stressed plants can be linked to higher plasticity of the cells and, therefore, better maintenance of the cell turgor. The formation and architecture of the cell wall are of great importance for signal transduction and stress sensing, so cell wall-related proteins can be linked to stress response (Le Gall et al. 2015).

### Response of potato genotypes to individual stresses: N deficiency (ND) and water deficiency (WD)

To investigate potential differences in the response to combined NWD stress compared to the single stress factors, we also analyzed the proteomic response of all four potato genotypes to individually applied ND and WD. The LC–MS runs were separately done for NWD/control and ND/WD/control. The proteome analysis of ND and WD samples allowed the identification of 699 proteins based on 2,354 non-conflicting peptides. Protein profiles were investigated using PCA plots (Suppl. Fig. S2) independently for ND and WD treatments. The four potato genotypes and the treatments clustered distinctly from the control in both, ND and WD treatments in experiment 1, a clear grouping was, however, not observed in experiment 2. In response to ND 38, 14, 5, and 29 DAPs were found in genotypes ‘Tomba’, ‘Kiebitz’, ‘Kolibri’, and ‘Eurostarch’, respectively. WD caused significant changes in abundance of 38, 7, 19, and 23 proteins in genotypes ‘Tomba’, ‘Kiebitz’, ‘Kolibri’, and ‘Eurostarch’, respectively. The relatively low number of DAPs was caused by the weak proteomic response of plants in experiment 2, resulting in a reduced overlap between experiments 1 and 2. Proteins responding to ND and WD are presented in Suppl. Tables S5 and S6.

**Fig. 7:**
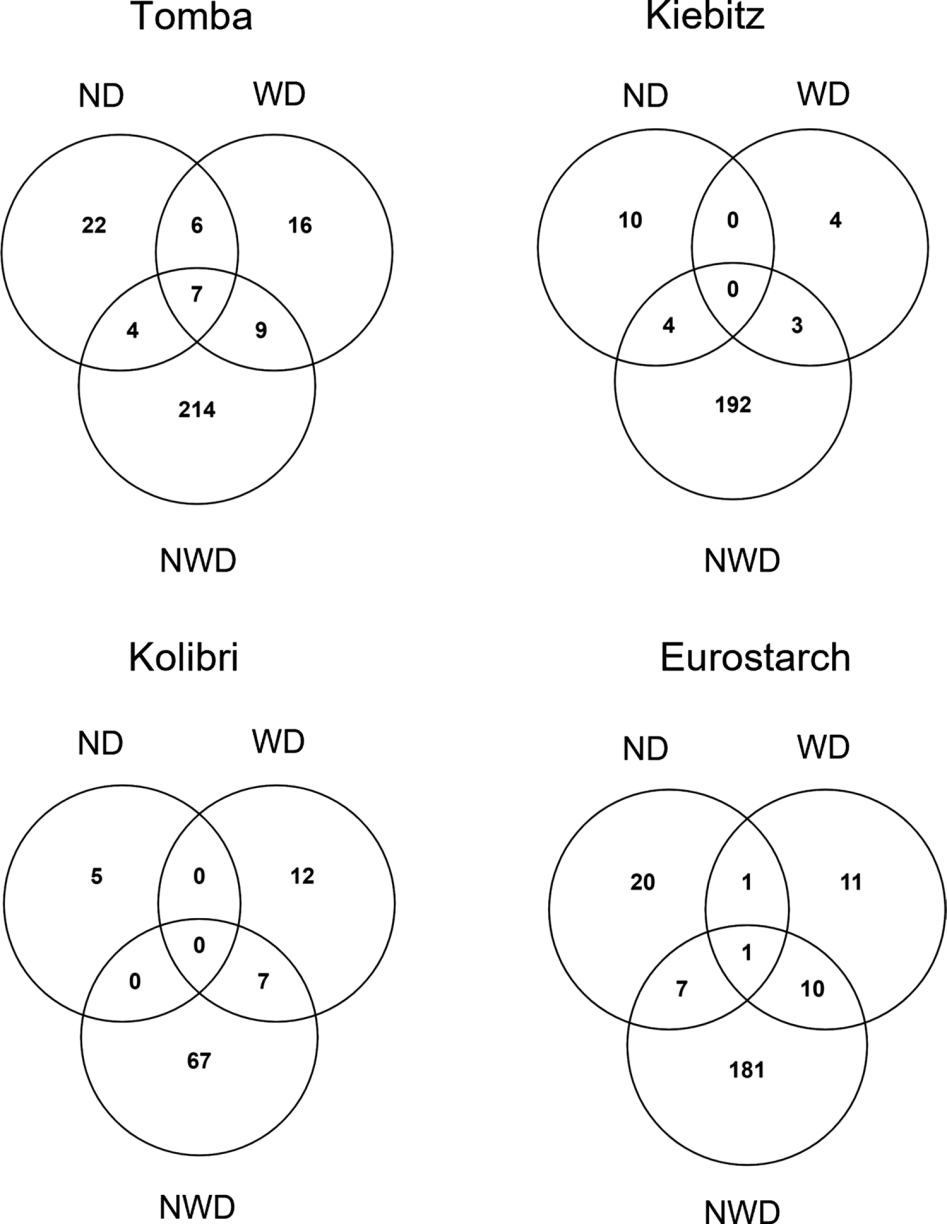
Number of differentially abundant proteins in the potato genotypes under ND, WD as well as combined NWD. The Venn diagrams show the number of proteins specific to examined conditions or shared between them. *ND* N deficiency; *WD* drought stress; *NWD* N and water deficiency

The purpose of analyzing the response to ND and WD was to find similarities and differences in the response of potato genotypes to NWD stress and individually applied stresses. This distinction between single and double stress is extremely important. Demirel et al. (2020) found differences in the regulation of biochemical pathways depending on the stress combination. Venn diagrams display the overlap of DAPs in the four potato genotypes in response to ND, WD, and NWD stresses (Fig. 7). For all genotypes, the highest number of DAPs was found for the NWD treatment, whereas much lower numbers were recorded under the single stresses and even fewer DAPs were detected in the overlaps. In Tables 2, 3, proteins overlapping and specific to ND, WD, and NWD are presented for the most contrasting genotypes ‘Tomba’ and ‘Kiebitz’, respectively. Proteins specific to NWD are not included in Tables 2, 3, this information is to be found in Suppl. Tables S2 and S3. The data for genotypes ‘Eurostarch’ and ‘Kolibri’ is presented in Suppl. Tables S7 and S8.

**Table 2:** List of differentially abundant proteins in the tolerant potato genotype ‘Tomba’ induced by N deficiency (ND), drought stress (WD) or combined N deficiency with drought stress (NWD)

Conditions	Accession	Protein description (according to Uniprot)	KEGG classification 2nd dimension	Ratio stress/control					
				Experiment 1			Experiment 2		
				ND	WD	NWD	ND	WD	NWD
ND/WD/NWD	400058564	3-hydroxy-3-methylglutaryl CoA synthase	Carbohydrate metabolism	0.51	0.53	0.29	0.59	0.53	0.34
ND/WD/NWD	400030650	Glycosyltransferase	Enzymes*	0.40	0.54	0.41	0.46	0.50	0.43
ND/WD/NWD	400030676	2-oxoglutarate-dependent dioxygenase	Enzymes*	0.49	0.40	0.35	0.44	0.41	0.24
ND/WD/NWD	400072701	S-adenosylmethionine synthetase	Amino acid metabolism	0.54	0.35	0.35	0.47	0.36	0.33
ND/WD/NWD	400041818	Phospho-2-dehydro-3-deoxyheptonate aldolase 2	Amino acid metabolism	0.53	0.46	0.65	0.40	0.48	0.46
ND/WD/NWD	400054532	Katanin p60 ATPase-containing subunit	Cell growth and death	0.49	0.44	0.46	0.34	0.47	0.08
ND/WD/NWD	400003666	UDP-glucose 6-dehydrogenase	Carbohydrate metabolism	0.50	0.58	0.54	0.55	0.44	0.59
ND/WD	400043112	Light-induced protein	Environmental adaptation*	2.10	1.86	1.26	1.55	1.51	1.86
ND/WD	400018192	Rhamnose biosynthetic enzyme 1-like	Carbohydrate metabolism	0.44	0.38	NA	0.39	0.43	NA
ND/WD	400023675	D-3-phosphoglycerate dehydrogenase	Amino acid metabolism	0.63	0.59	0.45	0.66	0.63	0.67
ND/WD	400053209	Early tobacco anther 1	Uncharacterized*	0.48	0.63	0.51	0.53	0.65	0.84
ND/WD	400003652	Heat shock protein	Chaperones and folding catalysts	0.66	2.71	1.09	0.66	2.05	0.55
ND/WD	400055488	Phenylalanine ammonia-lyase	Amino acid metabolism	0.49	0.24	NA	0.47	0.41	NA
ND/NWD	400035925	Vacuolar processing enzyme 1	Peptidases and inhibitors	2.50*	1.21	1.75*	1.94	1.01	5.11
ND/NWD	400008936	Oxidoreductase, 2OG-Fe(II) oxygenase family	Enzymes*	0.49	0.80	0.28	0.62	0.75	0.50
ND/NWD	400048984	Cysteine proteinase 3	Peptidases and inhibitors	1.68	0.75	0.66	2.59	0.79	1.66
ND/NWD	400007182	Dihydrolipoyl dehydrogenase	Carbohydrate metabolism	0.59	0.67	0.55	0.62	0.67**	0.65
WD/NWD	400031568	Granule-bound starch synthase 1	Carbohydrate metabolism	1.23	0.47	0.58	1.55	0.32	0.40
WD/NWD	400008351	Small heat shock protein	Folding, sorting and degradation	0.76	8.14	2.94	0.46	21.66	7.47
WD/NWD	400081247	Phospholipase A1	Lipid Metabolism	0.90	0.49	0.47	1.11	0.53	0.63
WD/NWD	400057147	Plastid-dividing ring protein	Chromosome and associated proteins	1.21	0.64	0.58	0.88	0.62	0.61
WD/NWD	400007216	Uncharacterized protein	Uncharacterized*	0.66	0.52	0.49	0.73	0.50	0.49
WD/NWD	400050256	Ribulose-phosphate 3-epimerase,	Carbohydrate metabolism	1.41	1.60	0.57	2.09	2.24	0.60
WD/NWD	400047146	S-adenosylmethionine synthetase 1	Amino acid metabolism	0.84	0.45	0.56	0.66	0.41	0.25
WD/NWD	400087679	S-adenosylmethionine synthetase 2	Amino acid metabolism	0.65	0.18	0.23	0.60**	0.24	0.25
WD/NWD	400078206	Tubulin beta chain	Cytoskeleton proteins	0.95	0.54	0.47	0.81	0.65	0.37
ND	400039851	Subtilisin-like protease preproenzyme	Folding, sorting and degradation/ Signaling molecules and interaction*	1.61	1.19	0.98	2.79	1.30	1.89
ND	400044209	Harpin binding protein 1	Environmental adaptation*	1.81	0.99	0.85	2.14	1.36	1.31
ND	400050664	Elongation factor 1-alpha	Translation	0.40	0.49	NA	0.59	1.35	NA
ND	400075611	Catalase isozyme 2	Carbohydrate metabolism	0.26	0.67	0.69	0.64	0.60	0.30
ND	400022085	Peptidyl-prolyl cis–trans isomerase	Chaperones and folding catalysts*	1.79	0.96	0.88	1.90	0.92	0.64
ND	400064434	Thioredoxin	Enzymes*	1.77	1.39	0.83	1.92	1.66	1.21
ND	400041576	Cinnamyl alcohol dehydrogenase	Biosynthesis of other secondary metabolites*	1.67	1.31	1.02	2.75	1.79	1.08
ND	400069750	Chloroplast sedoheptulose-1,7-bisphosphatase	Energy metabolism	1.70	0.81	0.94	1.79	1.27	1.19
ND	400050234	Geranylgeranyl reductase	Metabolism of cofactors and vitamins	0.43	0.72	0.76	0.38	0.60	0.45
ND	400057522	Suberization-associated anionic peroxidase	Enzymes*	1.68	0.95	0.90	2.11	1.43	2.16
ND	400044818	Glucose-6-phosphate 1-dehydrogenase	Carbohydrate metabolism	0.36	1.09	NA	0.36	0.91	NA
ND	400024090	Phosphoribulokinase	Energy metabolism	1.72	1.21	0.98	1.55	1.30	0.84
ND	400065504	Receptor protein kinase	Signal transduction*	2.03	0.71	NA	1.77	1.18	NA
ND	400000946	Arginine–tRNA ligase	Translation*	0.30	0.85	NA	0.51	1.25	NA
ND	400031351	Fructose-bisphosphate aldolase	Carbohydrate metabolism	1.51	0.82	0.85	1.77	1.03	0.81
ND	400057332	Fructose-bisphosphate aldolase	Carbohydrate metabolism	1.81	0.97	1.23	1.53	1.21	1.02
ND	400083971	Calmodulin-1	Signal transduction	1.81	1.18	0.74	1.83	1.33	1.37
ND	400081752	Uncharacterized protein	Uncharacterized*	1.53	0.99	1.04	2.27	1.37	1.89**
ND	400001149	Glycosyltransferase	Metabolism of terpenoids and polyketides	0.66	0.81	0.93	0.42	0.61	0.53
ND	400011133	Glutamine synthetase	Energy metabolism	1.51	0.92	NA	1.55	1.29	NA
ND	400039222	2-deoxyglucose-6-phosphate phosphatase	Carbohydrate metabolism	1.57	1.50	NA	1.68	1.31	NA
ND	400036729	U2 small nuclear ribonucleoprotein A	Transcription	0.64	1.23	NA	0.54	1.73	NA
WD	400004360	Ascorbate peroxidase	Carbohydrate metabolism	0.67	4.51	1.18	0.77	2.49	1.49
WD	400052308	CBS domain-containing protein	Uncharacterized*	0.65**	1.67	1.18	1.36	1.90	0.77
WD	400079161	Phospho-2-dehydro-3-deoxyheptonate aldolase 1	Amino acid metabolism	0.30	0.25	NA	0.20	0.26	NA
WD	400026271	Stigma expressed protein	Peptidases and inhibitors *	1.01	2.52	0.76	1.09	2.03	0.65**
WD	400071115	(S)-2-hydroxy-acid oxidase	Carbohydrate metabolism	0.90	1.70	1.18	1.35	1.51	0.92
WD	400003356	Granule-bound starch synthase 2	Carbohydrate metabolism	0.85	0.58	0.92	0.70	0.40	0.52
WD	400070986	Heat shock protein 70	Folding, sorting and degradation	1.72	4.29	NA	0.86	3.63	NA
WD	400011762	Invertase inhibitor	Carbohydrate metabolism*	0.74	3.02	NA	0.41	1.60	NA
WD	400021142	Class II small heat shock protein	Folding, sorting and degradation	0.87	11.26	2.89**	0.80	8.04	6.05
WD	400022265	Galactose mutarotase	Carbohydrate metabolism*	0.96	1.81	0.84	1.30	1.90	0.54
WD	400095387	Uncharacterized protein	Uncharacterized*	2.04	1.89	0.92	1.44	1.68	0.67
WD	400073479	Uncharacterized protein	Uncharacterized*	1.26	4.60	0.79	0.84	7.72	0.62
WD	400048880	DUF1995 domain-containing protein	Uncharacterized *	1.47	0.41	NA	0.97	0.48	NA
WD	400074842	Small rubber particle protein	Environmental adaptation*	0.81	1.77	0.99	1.45	2.18	0.93
WD	400055410	SBT1 protein	Folding, sorting and degradation/ Signaling molecules and interaction*	1.74	0.39	NA	1.20	0.36	NA
WD	400064274	Subtilisin-like protease	Folding, sorting and degradation/ Signaling molecules and interaction*	1.35	0.60	0.71	1.20	0.60	1.34

The abundance is presented in the form of fold change. Accession numbers are given without the PGSC003DMT prefix. Full details of the protein identification are stored together with raw data

*Classification performed manually, ** Fold change within significance limits, but *P* value higher than 0.05

**Table 3:** List of differentially abundant proteins in the sensitive potato genotype ‘Kiebitz’ induced by nitrogen deficiency (ND), drought stress (WD) or combined N deficiency with drought stress (NWD)

Conditions	Accession	Protein description (according to uniprot)	KEGG classification 2nd dimension	Ratio stress/control					
				Experiment 1			Experiment 2		
				ND	WD	NWD	ND	WD	NWD
ND/NWD	400035925	Vacuolar processing enzyme 1	Folding, sorting and degradation	5.41	1.34	7.84	2.76	1.90	4.25
ND/NWD	400029393	Plasma membrane polypeptide	Signal transduction*	4.67	1.03	1.54	3.31	2.13	1.74
ND/NWD	400057418	Glycerophosphodiester phosphodiesterase	Lipid metabolism*	1.74	0.90	1.88	1.74	1.15	1.71
ND/NWD	400048984	Cysteine proteinase 3	Folding, sorting and degradation	2.39	0.62	2.04	2.28	1.95**	1.51
WD/NWD	400058896	Aldehyde dehydrogenase (NAD( +))	Carbohydrate metabolism	1.42	1.60	2.14	1.49**	2.32	2.17
WD/NWD	400072701	S-adenosylmethionine synthase	Amino acid metabolism	0.75	0.56	0.43	0.51	0.36	0.32
WD/NWD	400078206	Tubulin beta chain	Cell growth and death	0.68	0.60	0.42	0.73	0.65	0.51
ND	400075915	Desacetoxyvindoline 4-hydroxylase	Biosynthesis of other secondary metabolites*	1.85	0.83	1.26	2.09	1.26	1.64
ND	400043112	Light-induced protein	Environmental adaptation*	2.50	2.83**	1.32	2.36	1.57**	2.22
ND	400044209	Harpin binding protein 1	Environmental adaptation*	1.90	1.06	1.46	1.76	1.24	1.41
ND	400070131	Carboxypeptidase	Folding, sorting and degradation	1.84	0.93	NA	1.78	0.90	NA
ND	400031568	Granule-bound starch synthase 1	Carbohydrate metabolism	2.45	0.71	0.65	2.02	0.35	0.76
ND	400064274	Subtilisin-like protease	Folding, sorting and degradation/ Signaling molecules and interaction*	1.66	0.75	1.55	1.60	0.92	0.97
ND	400025043	Pom14 protein	Membrane transport*	2.22	1.26	1.31	1.63	1.29	1.03
ND	400038370	3-beta hydroxysteroid dehydrogenase/somerase	Lipid metabolism*	2.35	1.29	NA	2.09	1.54	NA
ND	400065504	Receptor protein kinase	Signal transduction*	1.78	1.06	NA	1.75	1.37	NA
ND	400015365	ATP synthase subunit beta	Energy metabolism	1.73	1.10	0.86	1.52	0.88	1.03
WD	400083137	P5CDH1	Amino acid metabolism	1.10	1.58	0.44	1.01	1.90	0.77
WD	400003356	Granule-bound starch synthase 2	Carbohydrate metabolism	1.07	0.52	1.22	1.25	0.37	1.02
WD	400071822	RNA-binding protein	Uncharacterized*	0.71	0.51	NA	0.88	0.57	NA
WD	400006854	Cell division protein FtsZ	Chromosome and associated proteins	1.26	0.53	0.76	0.88	0.61	0.73

The abundance is presented in the form of fold change. Accession numbers are given without the PGSC003DMT prefix. Full details of the protein identification are stored together with raw data

*Classification performed manually, ** Fold change within significance limits, but *P* value higher than 0.05

While in genotype ‘Kiebitz’, no overlapping DAPs were identified between ND, WD, and NWD, in genotype ‘Tomba’, seven DAPs were shared between all three applied stresses (3-hydroxy-3-methylglutaryl CoA synthase, glycosyltransferase, 2-oxoglutarate-dependent dioxygenase, SAMS, phospho-2-dehydro-3-deoxyheptonate aldolase 2, katanin p60 ATPase-containing subunit, and UDP-glucose 6-dehydrogenase; Table 2). Those shared DAPs might indicate genotype-specific proteins for a general abiotic stress response. Especially 2-oxoglutarate-dependent dioxygenase and 3-hydroxy-3-methylglutaryl coenzyme A synthase are known to be part of an abiotic stress response (Meng et al. 2017; Tiwari et al. 2020a). Tiwari et al. (2020a) showed up- and down-regulation of 2-oxoglutarate-dependent dioxygenase to ND in roots and stolons of potato.

Four DAPs were overlapping for ND and NWD stress in genotype ‘Tomba’ (vacuolar processing enzyme (VPE) 1, oxidoreductase, cysteine proteinase 3, and dihydrolipoyl dehydrogenase) and nine for WD and NWD stress (e.g. granule-bound starch synthase 1, small heat shock protein, phospholipase A1, ribulose-phosphate 3-epimerase, and SAMS). Six proteins (light-induced protein, rhamnose biosynthetic enzyme 1-like, D-3-phosphoglycerate dehydrogenase, early tobacco anther 1, heat shock protein, and phenylalanine ammonia-lyase) were shared for ND and WD but were not responsive to NWD stress in genotype ‘Tomba’. In ‘Kiebitz’, four DAPs were common for ND and NWD (vacuolar processing enzyme 1, plasma membrane polypeptide, glycerophosphodiester phosphodiesterase, cysteine proteinase 3) and three for WD and NWD (aldehyde dehydrogenase (NAD( +)), SAMS and tubulin beta chain). The fact that VPE and cysteine proteinase 3 were also higher abundant in genotype ‘Kiebitz’ is supporting the idea of a general and not a tolerance-dependent stress response.

The DAPs for ND that appeared in the more tolerant variety ‘Tomba’ and the more sensitive variety ‘Kiebitz’ differed mainly in number. ‘Tomba’ (38 DAPs) had more DAPs overall than ‘Kiebitz’ (14 DAPs). The pathways affected were equivalent (e.g. carbohydrate metabolism, energy metabolism, environmental adaptation). Both genotypes shared a higher abundance of five DAPs (vacuolar processing enzyme 1, light-induced protein, hairpin binding protein 1, cysteine proteinase 3, and receptor protein kinase) under ND stress. What is striking, however, is that all DAPs of ‘Kiebitz’ were higher abundant in ND, while the ND treatment of ‘Tomba’ also showed some less abundant proteins. In combination with the morphological and physiological performance of ‘Tomba’, which included higher tuber and starch yield, this might display a faster adaptation of the genotype to the stress conditions (Dahal et al. 2019).

The genotype ‘Tomba’ showed several DAPs with a higher abundance in the WD treatment, especially in the functional class of chaperones (heat shock protein 70, class II small heat shock protein LE-HSP17.6), in environmental adaptation proteins (small rubber particle protein), and carbohydrate metabolism (invertase inhibitor, (S)-2-hydroxy-acid oxidase, ascorbate peroxidase). Interestingly, higher abundant HSPs under WD showed no longer a higher abundance under NWD. This suggests an influence of NWD on HSP biosynthesis. Whether the plant does not find sufficient resources to continue expressing heat shock proteins or whether the plant no longer needs those proteins in large quantities, remains unclear. Ascorbate peroxidase is part of a ROS-scavenging pathway in plants (Aghaei et al. 2009; Dahal et al. 2019).

## Conclusions

**Fig. 8:**
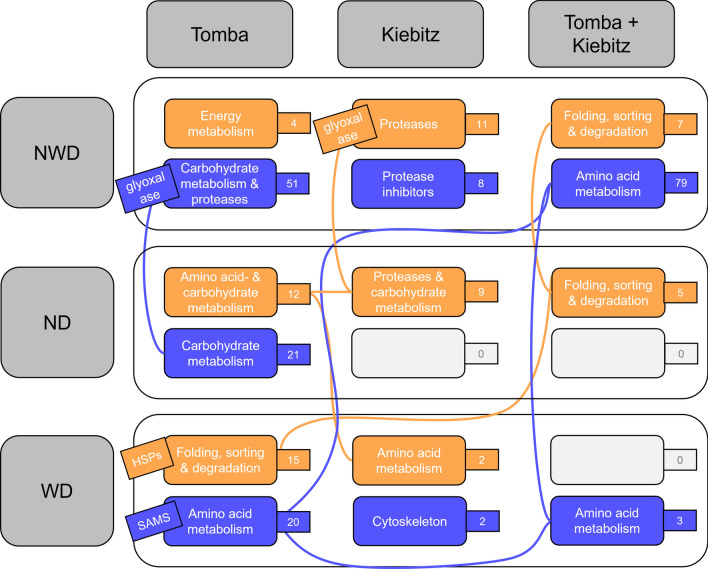
Overview of major changes in protein abundance after nitrogen deficiency (*ND*), water deficiency (*WD*) and combined stress (*NWD*) in the genotypes ‘Tomba’ and ‘Kiebitz’. The most important biochemical metabolic pathways are presented in the large tiles and the number of identified proteins in the small tiles. Orange: higher abundant proteins after stress. blue: lower abundant proteins after stress. Important individual proteins are indicated in the oblique tiles. Connecting lines indicate the same metabolic pathways in different variants. However, these do not necessarily contain the same proteins. Glyoxalase in ‘Kiebitz’ NWD was only significantly changed in abundance in one experiment

Potato genotypes grown under ND, WD and NWD displayed many common proteomic responses but also showed reactions specific for tolerant or sensitive genotypes, respectively (Fig. 8). (i) Increase of DAPs related to protein folding and decrease of amino acid metabolism participating DAPs was a general stress response to the combination of N deficiency and drought. (ii) Adaptions of the tolerant genotype ‘Tomba’ towards restructuring of the plant processes most likely led to a better NWD tolerance by higher abundance of DAPs participating in energy metabolism and a protease inhibitor, decrease of DAPs related to carbohydrate metabolism and proteases, and higher abundance of DAPs for amino acid and carbohydrate metabolism after ND. (iii) Proteins related to proteolysis were higher abundant in ‘Kiebitz’ suggesting that protein degradation was one of the key processes needed for plant survival under more severe stress. Upcoming studies need to be complemented by metabolic analyses related to the identified pathways (carbohydrate/energy and amino acid metabolism). The high abundance of a protease inhibitor in tolerant genotype ‘Tomba’ may be related to the overall better growth and less severe stress response of this genotype under NWD treatment. A possible explanation is that this genotype had already reduced proteolytic events at sampling. This hypothesis can be tested in follow-up studies applying a time-resolved sampling scheme. Collectively, our results suggest addressing the role of proteolytic events as a major focus in future functional studies.

The relatively low overlap of identified proteins when comparing the reaction to combined stress to responses to the single stresses rather drastically displays the need for test systems, which analyze double stressors on a broader scale for potatoes. This will be of grave importance in the future, when climate change, but also legal guidelines for fertilizer application, will lead to more challenging combinations of abiotic stresses.

### *Author contribution statement*

Material preparation, data collection, and analysis were performed by AMJ, KW, and CB. HPM, AS, SS, CB, and TW conceived and coordinated the project. The first draft of the manuscript was written by AMJ and KW. The manuscript was revised by CB, HPM, PM, AS, SS, and TW. All authors have read and approved the final document.

## Supplementary Information

Below is the link to the electronic supplementary material.

Supplementary file1 (DOCX 559 KB)

Supplementary file2 (XLSX 655 KB)

## Data Availability

The datasets generated during and analyzed during the current study are available in the IPK Gatersleben system e!DAL (Arend et al. 2014), available at: https://doi.org/10.5447/IPK/2023/4.

## Abbreviations

NWD: Nitrogen and water deficiency
ND: Nitrogen deficiency
WD: Water deficiency
DAP: Differentially abundant protein
SAMS: S-adenosyl-l-methionine synthase
