# Supplementary material for: Combined nitrogen and drought stress leads to overlapping and unique proteomic responses in potato
Source: Planta. 2023 Feb 16;257(3):58. doi: 10.1007/s00425-023-04085-4 (PMC9935667; doi:10.1007/s00425-023-04085-4)
Supplement: Supplementary file 1 — Supplementary file1 (DOCX 559 KB) [file 425_2023_4085_MOESM1_ESM.docx]

**Combined nitrogen and drought stress leads to overlapping and unique proteomic responses in potato**

Katharina Wellpott^1+^, Anna M. Jozefowicz^2+^, Philipp Meise^3^, Annegret Schum^3^, Sylvia Seddig^3^, Hans-Peter Mock^2,4^, Traud Winkelmann^1^, Christin Bündig^1^

^1^ Dept. Woody plant and propagation physiology, Institute of Horticultural Production Systems, Leibniz University Hannover, Herrenhäuser Str. 2, 30419 Hannover, Germany

^2^ Applied Biochemistry, Dept. Physiology and Cell Biology, Leibniz Institute of Plant Genetics and Crop Plant Research (IPK), OT Gatersleben, Corrensstr. 3, 06466 Seeland, Germany

^3^ [formerly] Institute for Resistance Research and Stress Tolerance, Julius-Kühn-Institute (JKI), Bundesforschungsinstitut für Kulturpflanzen, Rudolf-Schick-Platz 3a, 18190 Sanitz, Germany

^4^ Current address: Universidad de Costa Rica, CIGRAS, 11501-2060 San Pedro, Costa Rica

+ These authors have contributed equally to the manuscript.

Corresponding author:

Christin Bündig

Institute of Horticultural Production Systems

Leibniz Universität Hannover

Herrenhäuser Straße 2

30419 Hannover

Germany

Tel.: +49 (0) 511/7623239

Fax: +49 (0) 511/7623608

Email: buendig@baum.uni-hannover.de

ORCID: https://orcid.org/0000-0002-6280-1319


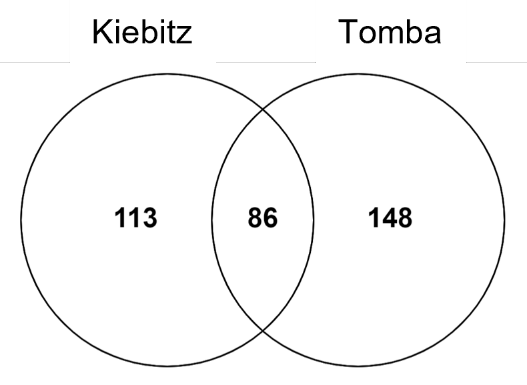


**Supplementary Fig. S1** Number of proteins differentially changed in both genotypes under NWD stress


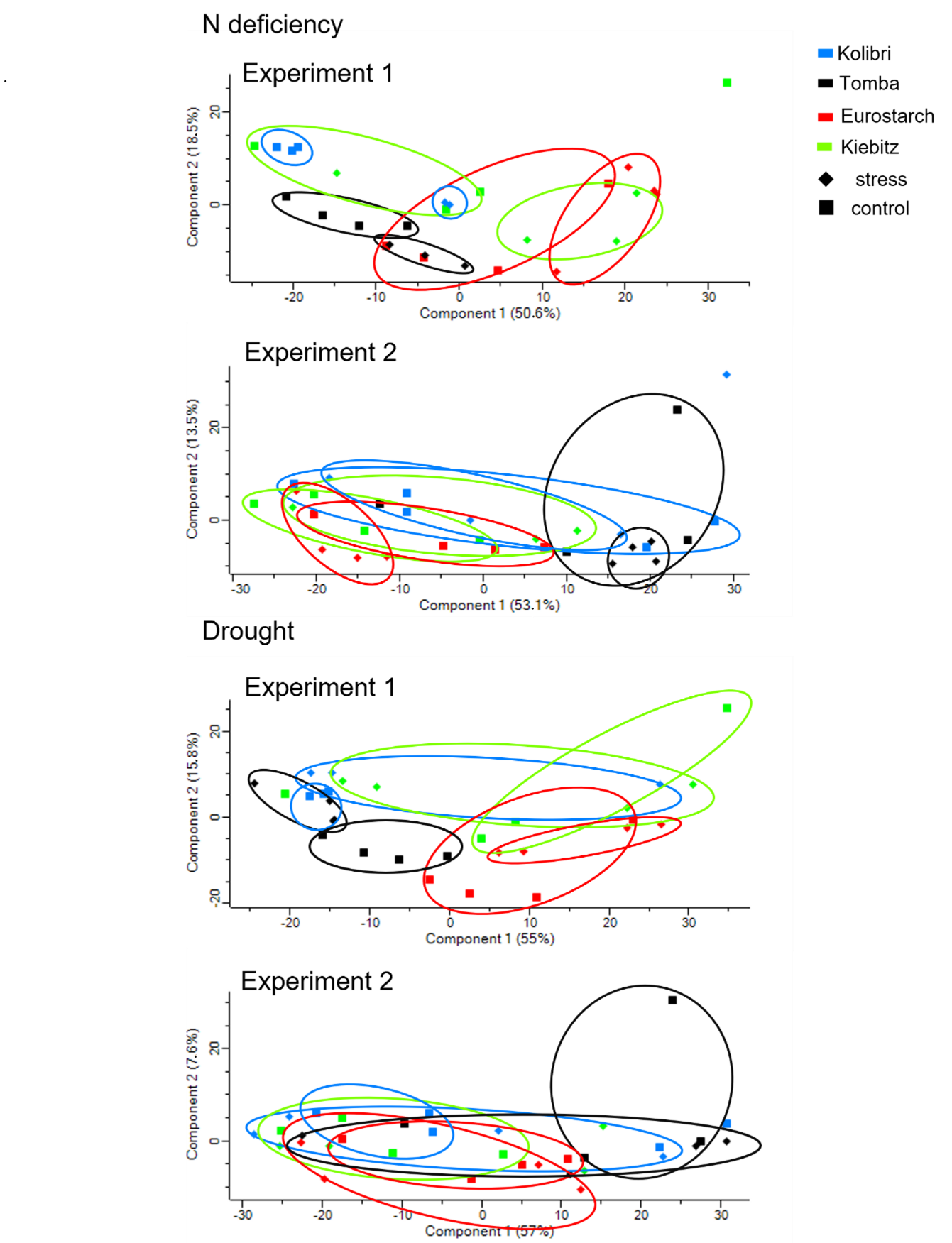


**Supplementary Fig. S2** Principal component analysis of differentially expressed proteins under N deficiency and drought conditions presented for both experiments separately. The shape of data points indicates stress (circle) or control conditions (square). All genotypes examined in the study are presented: Tomba (black), Kiebitz (green), Eurostarch (red), Kolibri (blue).
